# Influence of muscle fatigue on contractile twitch characteristics in persons with parkinson’s disease and older adults: A pilot study

**DOI:** 10.1016/j.prdoa.2021.100103

**Published:** 2021-08-08

**Authors:** Kelley G. Hammond, Mitchel A. Magrini, Jacob A. Siedlik, C. Scott Bickel, Marcas M. Bamman

**Affiliations:** a Department of Exercise Science and Pre-Health Professions, Creighton University, 2500 California Plaza, Omaha, NE 68104, USA; b Department of Physical Therapy, Samford University, 800 Lakeshore Pkwy, Birmingham, AL 35229, USA; c Dept of Cell, Developmental, and Integrative Biology, University of Alabama at Birmingham 1720 2^nd^ Ave South, Birmingham, AL 35294, USA; d Florida Institute for Human and Machine Cognition, 40 South Alcaniz St, Pensacola, FL 32502, USA

**Keywords:** Skeletal muscle, Rate of torque development, Evoked contraction, Fatigue

## Abstract

**Introduction:**

It is widely accepted that pathophysiological changes to the central nervous system of persons with Parkinson’s disease (PD) result in negative effects on motor function. However, less information is known regarding the pathology of PD on skeletal muscle. The purpose of this study was to determine the effect of a fatiguing isometric knee extension protocol on muscle mechanics using evoked twitch contractions in persons with PD and in non-impaired older adults (OLD).

**Methods:**

Evoked twitch contractions were examined during a fatiguing protocol in PD (66 ± 9 yr, n = 8) and OLD (65 ± 10 yr, n = 5). Participants performed 5-sec maximal isometric voluntary contractions of the quadriceps femoris with 5-sec rest for 3-min. Every 30-sec during rest intervals, a maximal transcutaneous electrical stimulus was administered to the quadriceps femoris to quantify evoked peak twitch torque (pTT), peak relaxation rate (pRR), and peak rate of torque development (pRTD).

**Results:**

A large effect of voluntary fatigue (%decline) was observed (*g* = 1.58). There were no significant differences in pTT (p = 0.09; 95% CI:-3.6, 0.28) or pRR (p = 0.11; 95% CI:-31, 3.6). However, the slope decline of pRTD in OLD (-35.4 ± 24.7) was greater than PD (-11.5 ± 11.4; p = 0.03), indicating that skeletal muscle in persons with PD is less fatigable compared to non-impaired older adults.

**Conclusion:**

The rate, not the maximum capacity, of torque generation of the muscle during a fatiguing knee extension protocol was affected by PD. Future studies are warranted to identify the mechanism(s) responsible for the observed differences in skeletal muscle contractile characteristics and potential myofiber distribution variation in PD.

## Highlights

• PD skeletal muscle contractile characteristics differ from normal aging.
• Evoked rate of torque development during fatiguing exercise is affected by PD.
• Peripheral adaptations to PD result in skeletal muscle fatigue-resistance.

## 1 Introduction

Parkinson’s disease (PD) is a neurodegenerative disease manifested as a progressive movement disorder with both non-motor symptoms and various combinations of four hallmark motor symptoms: tremor, muscle rigidity, bradykinesia, and postural instability. Degeneration of dopaminergic neurons leading to dopamine depletion in the midbrain basal ganglia, particularly the substantia nigra pars compacta, appears to initiate progressive motor dysfunction in PD [1]. The presentation of PD symptoms is accompanied by deficits in neuromuscular performance [2], [3], [4], [5], which may result in an increased risk of falls, loss of independence, and reduction in quality of life [6], [7].

Persons with PD display neuromuscular dysfunction distinct from normal aging that likely compounds the classically-described motor symptoms of the disease. Specifically, persons with PD generate force ~ 45% slower (i.e., rate of force development; RFD) [4], [8], have greater instability while producing force (i.e., less force steadiness) [9], and show differences in muscle activation while performing submaximal tasks compared to non-impaired older adults [10], [11], [12]. Studies that have examined muscle activation in PD have resulted in equivocal findings, with some reporting activation deficits [3], no difference in activation [4], and excessive activation [10] compared to healthy older adults. Moreover, it has been suggested that individuals with PD who demonstrate greater disability exhibit fatigue resistance across repeated contractions [3]. The divergence of research findings related to muscle activation could be attributed to differences in testing procedures; however, the degree to which the peripheral neuromuscular system contributes to the distinction between normal aging and PD remains poorly understood.

While it is challenging to differentiate age-related skeletal muscle degeneration and remodeling from PD-specfic changes to skeletal muscle in (older) persons with PD, motor unit reorganization resulting in differences in myofiber arrangement and size have been demonstrated [12]. In addition to age-related myofiber atrophy, it is evident that older adults and persons with PD experience varying degrees of myofiber loss, which may contribute to sarcopenia [13], [14], [15], [16]. Individuals with PD appear to undergo substantial motor unit reorganization leading to more extensive type I myofiber grouping than age-matched older adults [12]. Due to the fatigue-resistant qualities of type I myofibers, an increase in type I myofiber distribution and/or grouping may alter the response to repeated contractions (i.e., less decline in torque-generating properties).

The complexity of the neuromuscular system creates a challenge in distinguishing between centrally and peripherally-affected neuromuscular dysfunction in persons with PD due to peripheral changes in neural and/or contractile components of skeletal muscle. Therefore, the purpose of this study was to determine the effect of a fatiguing isometric knee extension protocol on skeletal muscle mechanics using neuromuscular electrical stimulation-evoked twitch contractions in persons with PD and in non-impaired older adults. Based upon previous research [3], [12], [17], we hypothesized that persons with PD would exhibit less fatigue throughout the isometric fatigue protocol compared to non-impaired older adults, as evidenced by a lower decline in peak twitch rate of torque development (pRTD), peak twitch torque (pTT), and twitch peak relaxation rate (pRR).

## 2 Methods

### 2.1 Human subjects

Thirteen individuals completed the testing protocol; eight persons with PD (M = 5, F = 3) and five non-impaired older adults (OLD; M = 4, F = 1). Participants were 55–79 yr of age and could ambulate ≥ 6 m independently. Additionally, PD participants were Hoehn and Yahr stage 2–3 and were medication stable for at least 4 wk. Individuals were excluded for secondary parkinsonism or Parkinson-plus syndromes; regular participation in resistance exercise training in the previous 6 mo; participation in drug studies or the use of investigational drugs within 30 d prior to screening; acute illness or active infection; confounding medical, neurological, or musculoskeletal conditions; or any known contraindication to exercise testing. The study was approved by The University of Alabama at Birmingham Institutional Review Board and each subject gave written informed consent prior to participation.

### 2.2 Medication profiles

A comprehensive medication history was collected during screening. All exercise testing and clinical evaluations were performed with the PD participants optimally medicated, in their best “on” state. Participants maintained their medication schedules during course of the study. Specific anti-parkinsonian medications and dosages varied widely among participants; thus, using the conversion factors of Tomlinson et al. [18], we computed the levodopa equivalent medication dosage (LED) for each participant to better standardize the data for a group summary. Among the seven PD participants reporting anti-parkinsonian medication usage, LED was 537 ± 356 mg/d (range 40–1001 mg/d); one PD participant was not taking dopaminergic medication.

### 2.3 Clinical assessments

All participants completed a series of clinical questionnaires and assessments including the Modified Baecke Physical Activity Questionnaire for Older Adults (MBQOA), Activity-specific Balance Confidence (ABC) Scale, and Fatigue Severity Scale (FSS). For the MBQOA and ABC questionnaires, a higher score indicates better function (i.e., higher daily activity or higher balance confidence); conversely, a lower FSS score is optimal, representing lower perceived fatigue severity. Additionally, the PD participants completed the 39-item Parkinson’s Disease Quality of Life Scale (PDQ-39) and were assessed for Section III (motor) of the Movement Disorder Society-sponsored revision of the Unified Parkinson’s Disease Rating Scale (MDS-UPDRS), as well as Hoehn & Yahr (H&Y) staging.

### 2.4 Repeated muscle contraction protocol

Participants performed a light warm-up at a submaximal, self-selected pace on a cycle ergometer for 5-min prior to being set up in the chair for the repeated muscle contraction protocol. Neuromuscular response to repeated muscle contractions was assessed using a test adapted from Callahan et al. [19] An isokinetic dynamometer (Biodex Medical, Shirley, NY) was used to measure unilateral maximum voluntary isometric contraction (MVIC) torque of the knee extensors on the more affected leg in PD and in the self-reported dominant leg in OLD [10]. Knee joint angle was standardized at 70° below horizontal, hip angle was standardized at 120°, and a safety belt was secured across the participant’s pelvis to secure them in the chair. The shank was secured to the Biodex lever arm two inches above the lateral malleolus. Participants were instructed to cross their arms over their chest during all isometric knee extension tasks. Familiziation of MVICs took place on a separate visit, and participants were required to repeat maximal effort for multiple MVICs during the familiarization session. For the repeated contractions protocol, participants were instructed to perform 5-sec MVICs alternated with 5-sec of rest for 3-min (18 contractions). The testers encouraged participants to kick against the lever arm of the dynamometer as hard and fast as possible and hold for 5-sec and were provided visual biofeedback of the torque-time signal during the protocol. Verbal cues were given in addition to an interval timer beep to instruct the participants when to contract and relax. Percent decline of mean torque (the plateau of the MVIC) was quantified as percent decline using the following equation: %decline = 100–100(MeanTorque_final_/ MeanTorque_initial_).

### 2.5 Evoked contractions

**Fig. 1:**
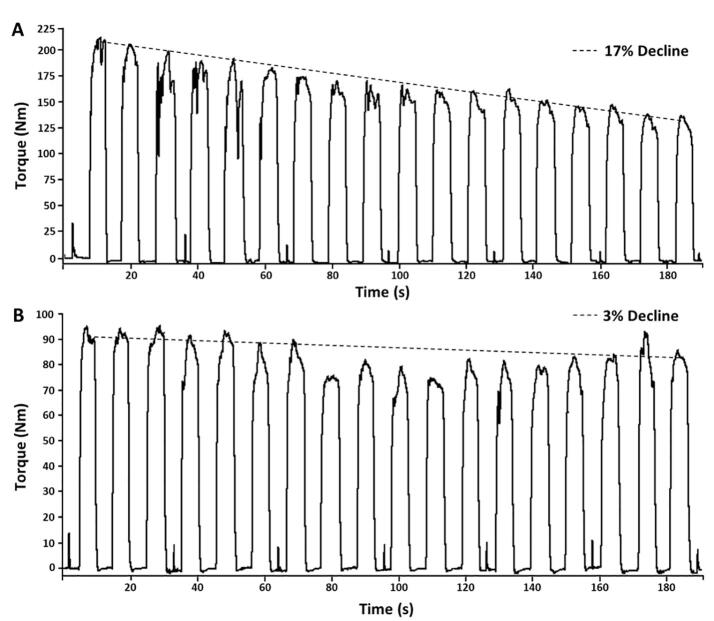
Methodological example of maximal voluntary contractions for representative OLD (A) and PD (B) participants with neuromuscular electrical stimulated twitches during the rest period every 30-sec (after every third contraction).

To better understand the peripheral contributions to neuromuscular dysfunction in PD, we investigated the peripheral component of the corticospinal pathway by using electrical stimulation to periodically induce maximal activation of the muscles. Transcutaneous electrical stimuli were delivered to the quadriceps femoris using an electrical stimulation unit (Grass S88 stimulator with a Grass Model SIU8T stimulus isolation unit, Grass Technologies, West Warwick, RI) via adhesive bipolar electrodes (Axelgaard PALS, 7x10 cm) over the distomedial and proximolateral quadriceps. Electrical stimulation was delivered pre-, during the rest period every 30-sec (after every third contraction), and post- during the repeated contraction protocol. Evoked contractions were elicited using a 200 µs [20] singlet pulse at individualized maximal parameters (Fig. 1). To determine the maximal parameters for stimulation, initial voltage for the singlet pulse started at 10 V. Maximal stimulation intensity was determined by increasing the intensity of the singlet pulse in 10 V increments until the elicited twitch failed to increase torque production. Maximal intensity was identified by real-time visual measurement confirmation of<5% change in peak twitch torque (pTT) with additional 10 V increase in stimulation intensity (voltage). The average maximal voltage required to obtain pTT was ranged from 110 to 130 V. pTT pRTD, and pRR were calculated from the evoked muscular contraction.

### 2.6 Signal processing

All torque signals were acquired simultaneously at 10 kHz via analog/digital conversion using a PowerLab 8/35 (ADInstruments, Bella Vista, Australia). LabChart Pro 8 (ADInstruments, Bella Vista, Australia) was used for application of pulses and data acquisition. Data were stored on a personal computer and processed off-line with a custom written analysis program (LabVIEW v. 19.0, National Instruments, Austin, TX, USA). All torque signals were zero-meaned, low-pass filtered using a zero-phase shift 4th-order Butterworth filter with a 15 Hz cutoff [21]. Six evoked contractions (twitches) over the 3-min repeated contraction protocol were individually analyzed. Each of the six evoked contractions were manually isolated via visual inspection and only these data were used to determine twitch properties. From these isolated evoked contractions, the peak torque (pTT; Nm) achieved during a 10-ms epoc (Kwon et al 2020) of each of the six evoked contractions during the repeated contraction protocol were calculated and used for analysis. Additionally, from each evoked contraction,the steepest positive 2.5-ms epoch slope (pRTD) prior to pTT and the steepest negative 2.5-ms epoch slope (pRR) following pTT of the torque-time curve (Nm·s) were automatically calculated and used for statistical analysis [22]. Because the variables of interest were peak values, force onset was not determined for each twitch contraction. Because the evoked contractions were visually inspected to exclude noise artifact, the use of onset detection methodologies was not necessary, as it would not change the steepest positive, steepest negative, or peak twitch torque values.

### 2.7 Statistical analysis

For each group, linear regressions were performed on the pRTD, pRR, and pTT changes during the fatigue protocol. Slopes and y-intercepts were retained for each linear regression model, and independent sample t-tests were used to identify significant differences in slopes and y-intercepts between the two groups (OLD vs. PD). Data analyzed via independent samples t-tests were found to be normally distributed except the slopes for pRTD, which were consequently analyzed using a Mann-Whitney test. Data are presented as mean differences (Mean_diff_) and 95% Confidence Intervals (CI) around those Mean_diff_. Due to the small samples size, effect sizes were calculated using Hedges’ *g* to determine the magnitude of differences between groups using pooled SD adjusted for sample size (*g = 0.15, small; 0.40, medium; 0.75, large*) [23], [24]. For descriptive purposes, dependent variables were analyzed using multilevel models with the lme4 package in R. Group was modeled as a fixed effect, while intercept and slope were modeled as random effects for each participant. Descriptive characteristics are reported as mean ± SD (Table 1). For all analyses, the level of significance was set at α = 0.05. All statistical analyses were performed using R, version 3.6.0.

**Table 1:** Descriptive characteristics for PD and OLD.

	PD	OLD
	n = 8	n = 5
Years Since Diagnosis	6 ± 8 (range 0–24 y)	–
Hoehn & Yahr	2.4 ± 0.4	–
MDS-UPDRS Section III	30 ± 9	–
PDQ-39 Mobility Sub-score	7 ± 5	–
PDQ-39 Total Index	5 ± 1	–
LED (mg/d)	537 ± 356	–
Sex	M = 5, F = 3	M = 4, F = 1
Age (y)	66 ± 9	65 ± 10
ABC (% confidence)	79 ± 7†	95 ± 5
Fatigue Severity Scale	4.1 ± 1.5*	2.2 ± 1.1
MBQOA (household)	1.6 ± 0.5	1.8 ± 0.3
MBQOA (sport)	0.8 ± 1.5	2.7 ± 2.9
MBQOA (leisure)	3.2 ± 3.7	6.0 ± 6.0
MBQOA (total)	5.7 ± 3.7	10.6 ± 5.5

MDS-UPDRS, Movement Disorders Society - Unified Parkinson’s Disease Rating

Scale; PDQ-39, 39-item Parkinson’s Disease Quality of Life Scale; LED, Levadopa

Equivalent Dose (mg/d); ABC, Activity-Specific Balance Confidence Scale; MBQOA,

Modified Baecke Physical Activity Questionnaire for Older Adults. *Different from CON, p < 0.05.

†Different from CON, p < 0.001. Values are mean ± SD.

## 3 Results

A large effect of percent decline in mean voluntary torque over the course of 18 voluntary contractions was observed (PD = 2.9% ± 20.8% (5.8 ± 22.0 Nm), OLD = 17.0% ± 14.6% (28.6 ± 27.9 Nm); p = 0.21; *g* = 1.58). There was no difference in initial voluntary torque between groups (PD = 119.8 ± 37.7 Nm, OLD = 149.1 ± 41.1 Nm; p = 0.21; *g* = 0.70).

**Fig. 2:**
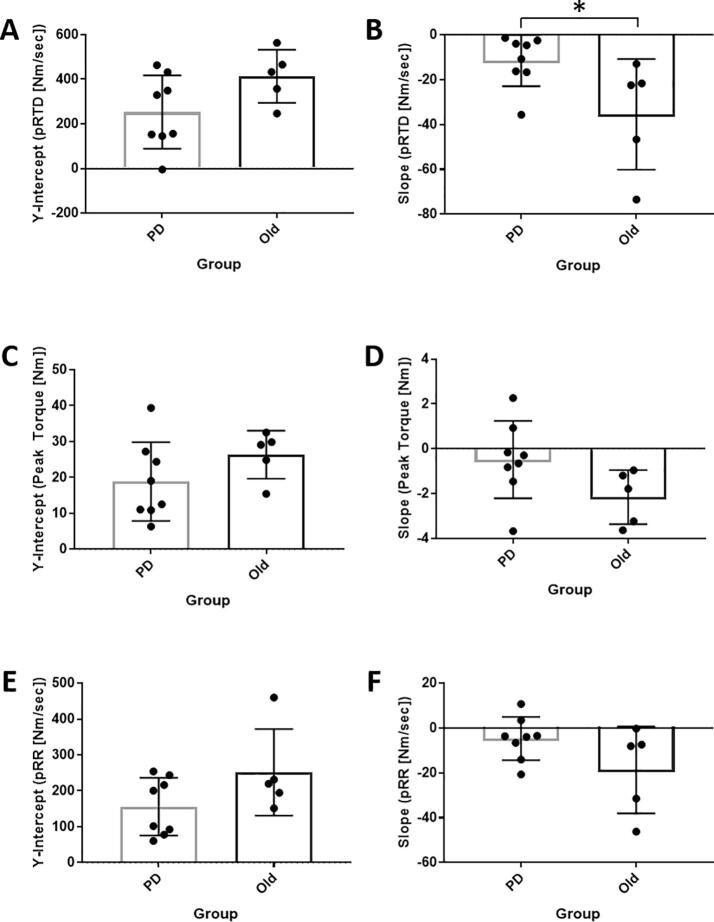
Slopes and y-intercepts linear regression models for pRTD (A-B), pTT (C-D), and pRR (E-F) in PD and OLD. *p ≤ 0.05.

**Fig. 3:**
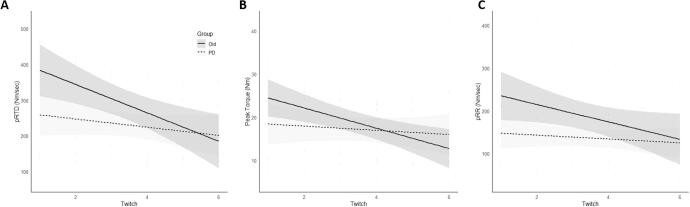
Predictive equations for evoked twitch characteristics during 3-min fatiguing protocol. (A) Peak rate of torque development (pRTD): ŷ = 352.26–44.05(PD) – 20.69(Twitch). 95% CI of Twitch = −32.4, −9. (B) Peak twitch torque (pTT): ŷ = 22.64–1.46(PD) – 1.12(Twitch). 95% CI of Twitch = −2.1, −0.15. (C) Peak relaxation rate (pRR): ŷ = 208.77–25.4(PD) – 10.26(Twitch). 95% CI of Twitch = −18.83, −1.66.

*pRTD.* There was no difference in the y-intercepts for pRTD between groups (Mean_diff_ = 160 ± 85 Nm/sec, 95% CI [-27, 347], p = 0.09; *g* = -2.25) (Fig. 2A). However, there was a significant difference in the slopes between groups (Mean_diff_: −24 ± 9.9 Nm/sec/twitch, 95% CI [-57, −5], p = 0.03; *g* = 2.99) (Fig. 2B). The multilevel model was developed for descriptive purposes (Fig. 3A).

*pTT.* There were no significant differences in the y-intercepts (Mean_diff_ = 7.5 ± 5.5 Nm, 95% CI [-4.6, 20], p = 0.2; *g* = -1.63) or slopes (Mean_diff_: −1.7 ± 0.89 Nm/twitch, 95% CI [-3.6, 0.28], p = 0.09; *g* = 2.26,) between groups (Fig. 2C-D). A descriptive multilevel model is shown in Fig. 3B.

*pRR.* There were no significant differences in the y-intercepts (Mean_diff_ = 95 ± 55 Nm/sec, 95% CI [-26, 217], p = 0.11; *g* = -2.07) or slopes (Mean_diff_: −14 ± 8 Nm/sec/twitch, 95% CI [-31, 3.6], p = 0.11; *g* = 2.09) between the two groups (Fig. 2E-F). A descriptive multilevel model is presented in Fig. 3C.

## 4 Discussion

These data provide novel information regarding the change in peripheral contractile characteristics in PD vs. OLD during a repeated contraction knee extension protocol and provide further insight into possible peripheral deficits in rapid force generation in PD. Specifically, the slope of evoked pRTD in PD across the repeated contraction protocoll was significantly less negative than OLD (p = 0.03), suggestive of PD-specific fatigue-resistance in evoked pRTD (Fig. 3A). However, no significant differences in evoked pTT or pRR were found between PD and OLD. These data suggest that the rate, not the maximum capacity, of force generation of the muscle during a repeated isometric knee extension protocol is affected by PD.

Although several studies have assessed pRTD and peak torque in patients with PD [3], [4], [8], [9], [17], few have examined the effect of a repeated isometric knee extension protocol on evoked muscle contractile characteristics in this population. In a cross-sectional comparison of persons with PD and healthy age- and sex-matched adults, Stevens-Lapsley et al. [3] measured voluntary isometric torque, central activation, and isokinetic fatigue of the quadriceps femoris. They divided the PD group into those with low-PD motor signs (UPDRS motor < 31.7) and high-PD motor signs (UPDRS motor ≥ 31.7); a higher UPDRS motor score is indicative of a greater degree of disability due to PD. The authors reported that individuals with high-PD motor signs experienced fatigue-resistance (i.e., less negative slope) in maximal voluntary torque production of the quadriceps muscles across repeated contractions, unlike healthy controls and those with low-PD motor signs. Stevens-Lapsley et al. [3] hypothesized that fatigue-resistance in PD may result from inadequate central drive to tax the muscle enough to affect metabolic fatigue. In agreement, we found that voluntary rapid force generation using evoked contractions was lower in PD vs. OLD, which could be indicative of skeletal muscle reorganization reflecting a greater distribution of type I muscle fibers. Together, the results of the current study and those of Stevens-lapsley et al.^3^ suggest that that physiological adaptations leading to fatigue resistance may manifest in the peripheral neuromuscular system.

Normal aging results in neuromuscular degeneration due to motor neuron death [25], [26], disruption of the neuromuscular junction (NMJ)[27], [28], and motor unit enlargement [29]. Motor neuron death and disruption of the NMJ results in motor unit reorganization, typically by axonal sprouting to reinnervate nearby denervated myofibers (i.e., motor unit enlargement). Type I myofiber distribution [10], as well as the prevalence of type I myofiber grouping [12], are both higher in PD and may possibly progress with PD progression. Type I motor units are not only recruited first and at a lower threshold during maximal voluntary contractions [30], but the myofibers within the motor units also possess a high oxidative capacity that facilitates fatigue resistance [31]. An increased type I myofiber distribution could explain the PD muscle’s lower capacity to generate torque rapidly and the reduction in fatigue in PD compared to OLD throughout the repeated contractions protocol observed in the current study. Therefore, based on the data from the current study, we hypothesize that a higher distribution of type I myofibers commonly observed in PD may have led to the reduced pRTD and increased fatigue-resistance (less decline in pRTD) in PD during a repeated contraction knee extension protocol compared to older adults (p = 0.03; Fig. 2B and 3A).

Neuromuscular remodeling that accompanies age-related motor neuron loss could be considered a compensatory attempt to mitigate loss of muscle mass and strength. However, while remodeling may help attenuate strength decline, the process may decrease RTD due to the increased distribution and grouping of type I myofibers. Of course, caution should be taken when interpreting these data due to limited sample size, and the current study should be reexamined with a larger sample to determine whether these non-significant findings are due to physiological adaptations to PD. The present study reports anti-parkinson medications as a levodopa equivalent dose (LED), which is widely used in the PD literature as part of describing the PD status of participants. However, the specific effects of these drugs on neuromuscular function have yet to be determined and this should be considered when interpreting our data.

In summary, individuals with PD better maintained pRTD throughout the repeated isometric knee extension protocol compared to OLD, but there were no differences in the percent declines in pTT or pRR between groups. The impact of repeated isometric contractions on these variables was distinct between groups—particularly in the ability to generate torque rapidly (pRTD) and we speculate this could be due to differences in myofiber type, distribution, and/or organization as we found in a separate PD vs. OLD cohort [12]. Although the results of this study using non-invasive techniques provide preliminary evidence of differences in contractile characteristics of the quadriceps femoris of PD and OLD, future studies using larger study cohorts and more invasive measures (i.e., skeletal muscle biospecimens) are warranted to determine direct relationships between muscle organization (i.e., myofiber type distribution and grouping) and force-generating characteristics in persons with PD. Additionally, forthcoming investigations assessing the influence of common anti-parkinson medications on peripheral neuromuscular function should be explored to provide a better understanding of the drug-specific effects.

## CRediT authorship contribution statement

**Kelley G. Hammond:** Conceptualization, Data curation, Formal analysis, Funding acquisition, Investigation, Methodology, Project administration, Resources, Software, Supervision, Validation, Visualization, Writing - original draft, Writing - review & editing. **Mitchel A. Magrini:** Conceptualization, Formal analysis, Methodology, Software, Supervision, Validation, Visualization, Writing - original draft, Writing - review & editing. **Jacob A. Siedlik:** Formal analysis, Methodology, Software, Visualization, Writing - original draft, Writing - review & editing. **C. Scott Bickel:** Conceptualization, Data curation, Methodology, Resources, Software, Writing - review & editing. Marcas M. Bamman - Funding acquisition, Investigation, Methodology, Project administration, Resources, Software, Supervision, Writing - review & editing.

## Declaration of Competing Interest

The authors declare that they have no known competing financial interests or personal relationships that could have appeared to influence the work reported in this paper.
